# Revision of *Nagiella* Munroe (Lepidoptera, Crambidae), with the description of a new species from China

**DOI:** 10.3897/zookeys.964.55703

**Published:** 2020-08-27

**Authors:** Xiao-Qiang Lu, Xi-Cui Du

**Affiliations:** 1 College of Plant Protection, Southwest University, Chongqing, China Southwest University Chongqing China

**Keywords:** DNA barcodes, Maximum Likelihood analysis, morphology, Pyraloidea, Spilomelinae

## Abstract

The genus *Nagiella* was studied using morphological and DNA barcode data. *Nagiella bispina***sp. nov.** is described as a new species, and *N. hortulatoides* Munroe is recorded in China for the first time. The diagnosis of this genus is revised, and the genitalia description of *N. quadrimaculalis* (Kollar and Redtenbacher) and *N. inferior* (Hampson) are given in English for the first time. *Nosophora incomitata* (Swinhoe) **stat. rev.** is removed from the synonym of *N. quadrimaculalis*. Photographs of the habitus and genitalia as well as COI DNA Barcode data of these four species are provided.

## Introduction

*Nagiella* Munroe, 1976 is the objective replacement name of *Nagia* Walker, 1866, with *N. desmialis* Walker, 1866 as the type species. Swinhoe (1894) described two species of *Nagia* and mentioned that *Nagia quadrimaculalis* (Kollar & Redtenbacher, 1844) = *Nagia desmialis*. However, Hampson (1899) regarded *Nagia* as a synonym of *Syllepte* Hübner, 1823 and his opinion was followed by some researchers (Shibuya 1928, 1929; Klima 1931, 1939a). Munroe (1976) proposed that *Nagiella* and *Syllepte* were different in genitalia and type of maculation and mentioned that the type species, *N. desmialis*, was generally considered a synonym of *Scopula quadrimaculalis*. Munroe’s opinion was followed by some researchers (Kirti and Sodhi 2001; Rose 2002; Ullah et al. 2017). In addition, *Nagiella* was regarded as a synonym of *Pleuroptya* Meyrick, 1890 (= *Patania* Moore, 1888) (Kirpichnikova 1987; Leraut 1997), and *Scopula quadrimaculalis* and *Sylepta inferior* were placed in *Pleuroptya* for a long time (Inoue 1982; Wang and Speidel 2000; Bae et al. 2008; Du 2009; Heppner 2012; Sasaki and Yamanaka 2013). Ullah et al. (2017) regarded *Nagiella* as a valid genus and published one cryptic species of it. Mally et al. (2019) placed *Nagiella* in Agroterini Acloque, 1897 based on morphological characteristics.

To date, four species of *Nagiella* have been identified worldwide, and they have been recorded in the Palaearctic and Oriental realms. These species are all distributed in China, with *N. hortulatoides* Munroe, 1976 being recorded in China for the first time in this study. *Nagiella inferior* and *N. quadrimaculalis* are widely distributed in the Palaearctic and Oriental realms (Wang 1980; Inoue 1982; Bae et al. 2008; Du 2009; Sasaki and Yamanaka 2013), with the latter species also recorded from Central Africa (Ghesquière 1942). In addition to China, *N. hortulatoides* is distributed in Myanmar. *Nagiella occultalis* Misbah & Yang in Ullah et al. 2017 is only distributed in China (Ullah et al. 2017). In this study, one new species, *Nagiella bispina*, is described based on morphological and DNA barcode data, and the diagnosis of this genus is revised.

## Materials and methods

### Taxon sampling

The specimens were collected by light trap at night and killed by ethyl acetate or ammonium hydroxide. The specimens are deposited in the College of Plant Protection, Southwest University, Chongqing, China (SWUCPP) and the Institute of Zoology, Chinese Academy of Sciences, Beijing (IOZ). Information on the specimens from which the DNA Barcode region of the COI gene was sequenced is shown in Table 1. In total, 24 sequences were analysed in this study, with eight being from the BOLD database (Ratnasingham and Hebert 2007; http://v4.boldsystems.org/). The sequences obtained from our laboratory have been uploaded to BOLD.

Genitalia preparation mainly follows Li and Zheng (1996). Images of the adults were captured with a digital camera (Nikon P7700), and images of the genitalia were captured with a digital camera (Leica DFC 450) attached to a digital microscope (Leica M205 A).

### DNA extraction, PCR amplification, and sequencing

In total, all five species of *Nagiella* were included for PCR analysis and DNA sequencing (Table 1). Total DNA from legs of fresh or dry specimens was extracted using the TIANGEN DNA Kit following the manufacturer’s instructions, and the 658-base pair (bp) barcode region of COI was amplified using the LepF1/LepR1 primers (Hajibabaei et al. 2006). PCR products were sent to Sangon Biotechnology Co., Ltd. (Shanghai, China) for sequencing using the aforementioned primers.

**Table 1.** Sample information for the *Nagiella* and outgroup specimens included in the study.

Species	Sequence ID	Location (China)	BOLD Accession NO. er
*N. hortulatoides* Munroe, 1976	LXQ180100	Yunnan	DULU001-19
	LXQ180099	Yunnan	DULU002-19
	LXQ180217	Yunnan	DULU003-19
*N. inferior* (Hampson, 1899)	LXQ180251	Hubei	DULU004-19
	LXQ180127	Yunnan	DULU005-19
	Pyr000509	Shanxi	CNPYD509-10
	Pyr000508	Shanxi	CNPYD508-10
*N. quadrimaculalis* (Kollar & Redtenbacher, 1844)	XD1405327	Sichuan	GBMIN79565-17
	XD1402131	Hainan	DULU006-19
	XD1402129	Hubei	DULU007-19
	Pyr002264	Shaanxi	CNPYB413-16
	Pyr002266	Shaanxi	CNPYB415-16
	Pyr000498	Hubei	CNPYD498-10
*N. occultalis* Misbah & Yang in Ullah et al. 2017	Pyr002290	Shaanxi	CNPYB439-16
	Pyr002397	Shaanxi	CNPYB407-16
	Pyr000499	Hubei	CNPYD499-10
*N. bispina* sp. nov.	LXQ180091	Guangdong	DULU008-19
	LXQ180092	Guangdong	DULU009-19
*Patania balteata* (Fabricius, 1798)	XD1405399	Sichuan	GBGL38467-19
	XD1405300	Sichuan	GBMIN79548-17
	XD1405441	Sichuan	GBGL38468-19
*P. chlorophanta* (Butler, 1878)	XD1404265	Guangxi	GBMIN79550-17
	XD1404239	Guangxi	GBMIN79549-17
	XD1401035	Guangxi	GBMIN79551-17

### Data analysis

All COI sequences were aligned by MEGA 7.0 (Kumar et al. 2016) and adjusted visually after being translated into amino acid sequences. Intraspecific and interspecific genetic divergence values were quantified based on the Kimura 2-parameter (K2P) distance model (Kimura 1980). Phylogenetic analysis was performed based on Maximum Likelihood (ML) with the GTR GAMMA model of nucleotide substitution, and with 1000 bootstrap replicates (Stamatakis et al. 2008). *Patania balteata* and *P. chlorophanta* were chosen as the outgroup species as they were members of the same tribe (Agroterini), but not congeneric with *Nagiella*.

## Results

### DNA sequence analysis

Overall, 24 COI sequences, including six of the outgroup species, were analysed. The dataset contained no obvious pseudogenes, indicating the correct target gene sequence was amplified and sequenced.

**Figure 1.**
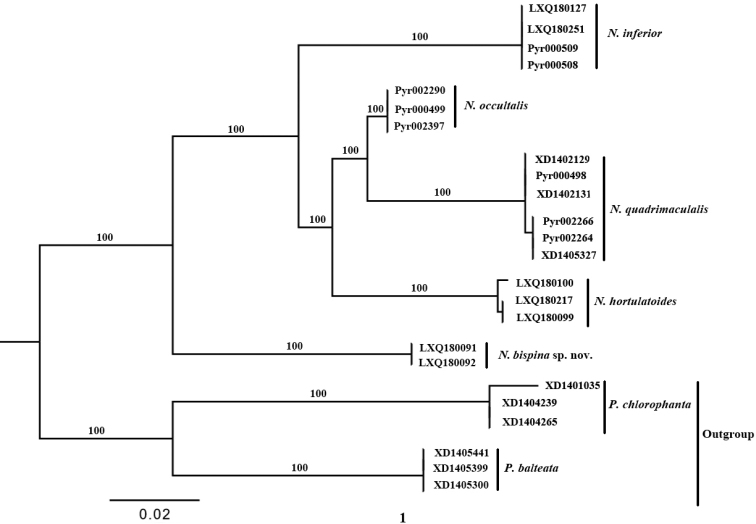
Phylogenetic hypothesis of relationships among five species of *Nagiella* inferred from a Maximum likelihood (ML) analysis of the DNA barcode data, with *Patania balteata* and *P. chlorophanta* as outgroup species.

Five monophyletic clades for *Nagiella* were observed in the resulting phylogenetic tree (Fig. 1). The pairwise genetic distances within and between these lineages are given in Table 2. The average intraspecific genetic distance ranged from 0.00 to 0.02%, while the average interspecific genetic distance ranged from 3.30 to 9.46%. The maximum intraspecific COI genetic distance was much less than the minimum interspecific distance. The monophyla observed in the phylogenetic analysis were in full congruence with our morphological hypotheses for the investigated species (Fig. 1).

**Table 2.** Kimura 2-parameter genetic distances in percent, calculated within (in bold) and between species of *Nagiella*.

	1	2	3	4	5
1 *N. hortulatoides* (*N* = 3)	**0.20**				
2 *N. inferior* (*N* = 4)	6.87	**0.00**			
3 *N. quadrimaculalis* (*N* = 6)	4.87	6.68	**0.09**		
4 *N. occultalis* (*N* = 3)	4.00	5.16	3.30	**0.00**	
5 *N. bispina* sp. nov. (*N* = 2)	8.95	9.46	8.80	7.57	**0.00**

### Taxonomy

#### Nagiella

Taxon classificationAnimaliaLepidopteraCrambidae

Munroe, 1976

5C02C8B4-4B15-5A91-B9A0-C0197D87219E

Nagia Walker, 1866: 1320 (preocc.). Type species: Nagia desmialis Walker, 1866, by monotypy.
Nagiella Munroe, 1976: 876. Type species: Nagia desmialis Walker, 1866, by monotypy (of Nagia Walker, 1866).

##### Diagnosis.

**Figures 2, 3.**
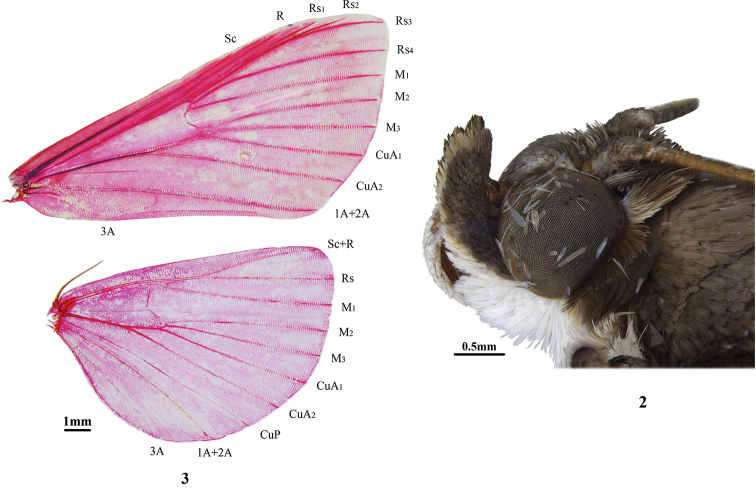
Head and wing venation of *Nagiella quadrimaculalis* (Kollar & Redtenbacher, 1844). Wing slide no. LXQ20001, male.

Frons rounded. Labial palpus broad, obliquely upturned and curved, compressed, third joint extremely minute, short and stout (Fig. 2). Male antenna with ventral cilia. Legs smooth. Fore wings near rectangular at the tips; length of cell approximately half of wing; R from cell at approximately two-thirds; Rs_2_ anastomosed with Rs_3_ approximately three-fifths beyond cell; Rs_1_ closely approximated to Rs_2_+Rs_3_; Rs_4_ curved towards Rs_2_+Rs_3_ at base; discocellulars arcuately incurved; M_2_, M_3_ and CuA_1_ from posterior angle of the cell uniformly at the base; CuA_2_ from three-fourths below the cell. Hindwing with length of cell one-third of wing; Sc+R anastomosed with Rs approximately one-fourth beyond the cell; M_2_, M_3_ and CuA_1_ separately from posterior angle of the cell; CuA_2_ from two-thirds below the cell; discocellulars incurved (Fig. 3). Male genitalia: Uncus short and wide; gnathos present in most species; valva lingulate, posterior margin with long setae cluster in most species; clasper near base, developed and pointed to sacculus; phallus cylindrical, cornuti absent in most species. Female genitalia: Apophyses anteriores longer than apophyses posteriores, rhomboidally expanded near base; ductus seminalis from the ductus bursae; corpus bursae oval, with signum.

##### Remarks.

According to Munroe (1976) and Ullah et al. (2017), *Nagiella* can be differentiated from its similar genera by its short and wide uncus, developed gnathos, broader valva with stout setae subapically, large oblique clasper and absence of cornuti, as well as by the type of wing maculation. In *N. bispina* sp. nov., however, the gnathos is absent, the valva costa has no stout setae, and cornuti are present. Other morphological and DNA barcode data of this new species indicate it as a member of the genus. Therefore, the diagnosis of *Nagiella* was revised based on previous studies and our research, including supplementation of the wing venation.

### Key to species of *Nagiella* based on morphology and genitalia

**Table:** 

1	Wings white, forewing with discoidal spot round	***N. hortulatoides***
–	Wings brown, forewing with discoidal spot squarish	**2**
2	Gnathos absent, phallus with a hook-shaped cornutus	***N. bispina* sp. nov.**
–	Gnathos present, phallus without cornutus	**3**
3	Uncus with setae on distal half; gnathos stubby, finger-like or tuberculiform	***N. inferior***
–	Uncus without setae; gnathos slender, finger-like	**4**
4	Forewing with white spot between orbicular spot and discoidal spot proportionally narrowed or elongate; uncus with distal margin slightly concave	***N. occultalis***
–	Forewing with white spot between orbicular spot and discoidal spot nearly square or rectangular; uncus with distal margin truncate	***N. quadrimaculalis***


#### Nagiella hortulatoides

Taxon classificationAnimaliaLepidopteraCrambidae

Munroe, 1976

C683F2D1-880B-57AB-B366-9C73D10C46BD

Figures 4 , 9

Nagiella hortulatoides Munroe, 1976: 876, figs 2, 14, 19.

##### Material examined.

China, Yunnan: 10 ♂♂, Honghe Prefecture, Huanglian Mountain, 900 m, 27.V.2018, leg. Xiao-Qiang Lu & Xi-Cui Du. Genitalia slide no.: LXQ18170 ♂, LXQ18187 ♂, LXQ18311 ♂.

##### Diagnosis.

**Figures 4–8.**
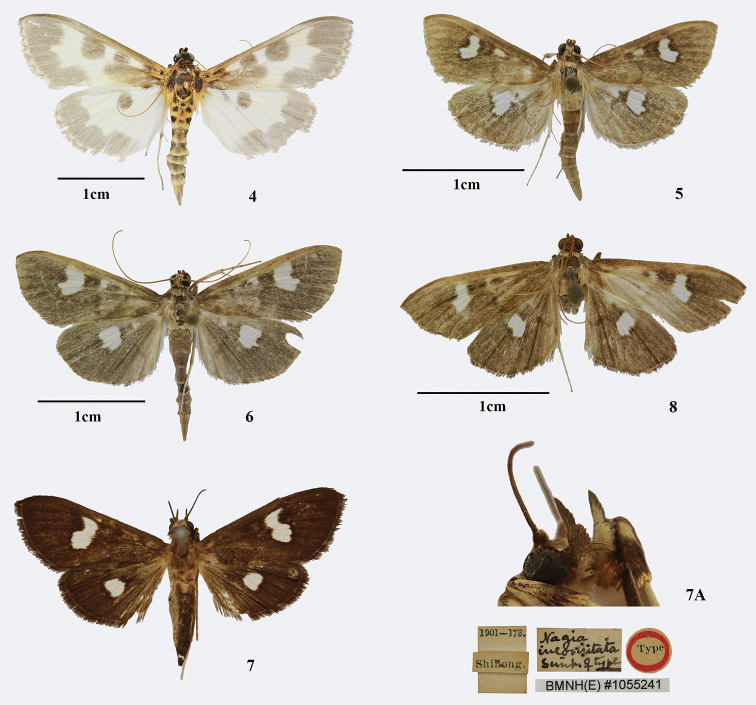
Habitus of *Nagiella* species **4***N. hortulatoides* male **5***N. inferior* male **6***N. quadrimaculalis* male **7***Nagia incomitata*Swinhoe 1894 female, type, BMNH Pyr., London. **7A** head **8***Nagiella bispina* sp. nov. male, holotype.

**Figures 9–15.**
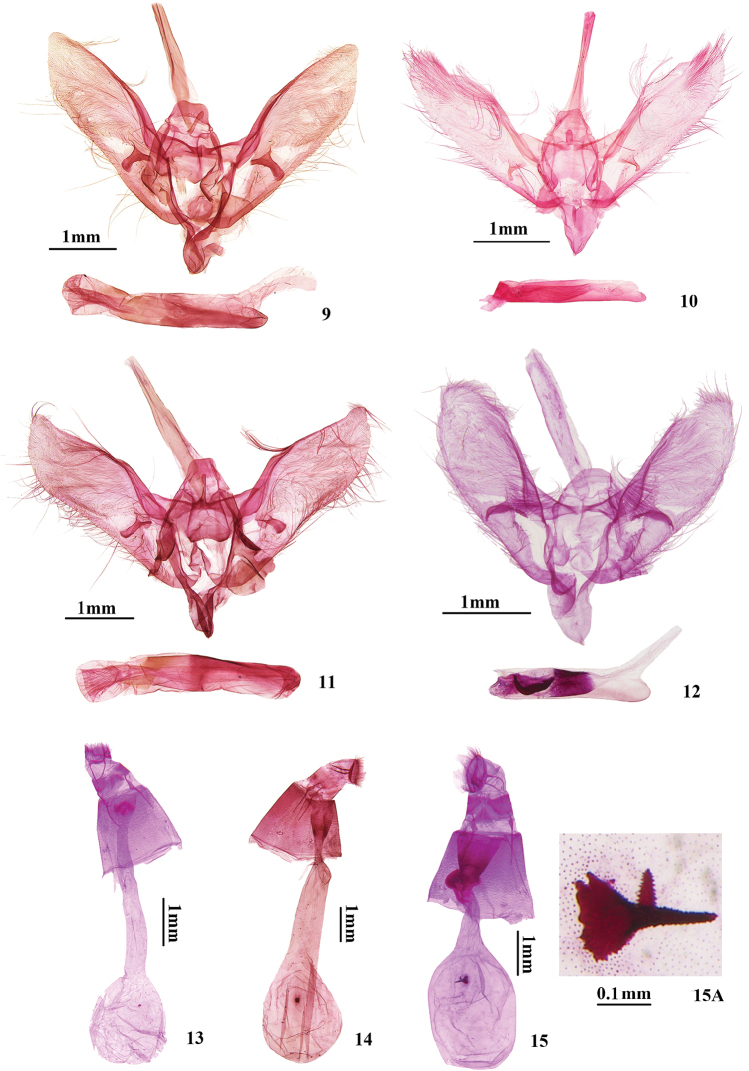
Genitalia of *Nagiella* species **9***N. hortulatoides*: male, genitalia slide no. LXQ18311 **10, 13***N. inferior*: **10** male, genitalia slide no. LXQ18291 **13** female, genitalia slide no. XLJ14239 **11, 14***N. quadrimaculalis*: **11** male, genitalia slide no. LXQ18310 **14** female, genitalia slide no. LXQ18306 **12, 15***N. bispina* sp. nov.: **12** male, holotype, genitalia slide no. XLJ14011 **15** female, paratype, slide no. XLJ14009 **15A** signa.

Adult (Fig. 4): Frons, palpi, basal antenna, most of vertex black. Thorax orange with blackish-fuscous spot. Wings white, light orange at base, maculation grey, with terminal line white, discontinuous. Forewing with orbicular spot and discoidal spot round, a large elongate elliptical spot from base to orbicular spot below cell; grey terminal area broad, with inside concave between M_1_ and CuA_2_. Hindwing with discoidal spot round; grey terminal area broad, with inside slightly concave between M_2_ and CuA_2_. Abdomen with first and second segment orange with three black spots, the rest grey. Male genitalia (Fig. 9): Uncus trapezoidal. Gnathos slender, finger-like. Valve elongate lingulate, posterior margin with clusters of long setae near middle and terminal, clasper thickly finger-like. Female genitalia: Corpus bursae with a round signum (Munroe 1976).

##### Distribution.

China (Yunnan), Myanmar (Munroe 1976).

##### Remarks.

This species is recorded for the first time in China.

#### Nagiella inferior

Taxon classificationAnimaliaLepidopteraCrambidae

(Hampson, 1899)

77EA19A4-157C-5492-AD85-977D38A92064

Figures 5 , 10 , 13

Sylepta [sic] inferior Hampson, 1899: 724.
Botys quadrimaculalis Motschulsky, 1861: 37.
Nagiella inferior : Munroe, 1976: 876.
Pleuroptya inferior : Inoue, 1982: 343.

##### Material examined.

China, Liaoning: Huanren County, Laotuding, 28.VII.2012, leg. Dan-Dan Zhang & Li-Jun Yang (SYSU); Gansu: 1 ♂, Kangxian County, Baiyun Mountain, 1200 m, 3.VII.2018, leg. Xiao-Qiang Lu & Xi-Cui Du; Shanxi: 1 ♂, Jincheng City, Manghe, 725 m, 28.VI.2018, leg. Xiao-Qiang Lu & Xi-Cui Du; Shaanxi: Ningxia County, Xunyangba Town, 1400 m, 4.VIII.2014, leg. Jiu-Yang Luo & Kai-Li Liu; 3 ♂♂, Taibai County, Huangbaiyuan Town, 1200 m, 19.VIII.2014, leg. Kai-Li Liu; 6 ♂♂, 1 ♀, Baojilong County, 900 m, 6.VII.2018, leg. Xiao-Qiang Lu & Xi-Cui Du; Hubei: 15 ♂♂, Dabie Mountain, Taohua Village, 590 m, 25–28.VI.2014, leg. Li-Jun Xu; 2 ♂♂, Xiangyang City, Maqian Town, 1100 m, 19.VI.2018, leg. Xiao-Qiang Lu & Xi-Cui Du; Zhejiang: 1 ♂, Jiulong Mountain, 50 m, 4.VIII.2011, leg. Xiao-Bing Fu; 9 ♂♂, Tianmu Mountain Nature Reserve, 400 m, 25–28.VII.2011, leg. Xi-Cui Du & Xiao-Bing Fu; 4 ♂♂, Qingliangfeng Nature Reserve, 300 m, 18–22.V.2012, leg. Xiao-Bing Fu; Tibet: Motuo County, Didong Village, 840 m, 15.VIII.2006, leg. Fu-Qiang Chen (IOZ); Chongqing: 1 ♂ Jingfoshan Nature Reserve, 679 m, 15.IX.2018, leg. Xi-Cui Du; 1 ♂, Hechuan Farm, 230 m, 3.VII.2009, leg. Xi-Cui Du; 6 ♂♂, Chengkou County, Dongan Village, xingtian Village, 1300 m, 26.VI.2013, leg. Gui-Qing He & Li-Jun Xu; Sichaun: 4 ♂♂, Tongjiang County, Nuoshui River Scenic Area, 700 m, 5.VII.2013, leg. Gui-Qing He & Dan Xu; 1 ♂, Nanjiang County, Guangwu Mountain, 900 m, 10.VII.2013, leg. Gui-Qing He & Li-Jun Xu; 1 ♂, 2 ♀♀, Huagaoxi Nature Reserve, Guandou Village, 763 m, 11.X.2014, leg. Li-Jun Xu & Dan Xu; Guizhou: 1 ♂, Kuankuoshui, Baishao, 800 m, 12.VIII.2010, leg. Xi-Cui Du; 1 ♂, Maolan Nature Reserve, Lanei Village, 806 m, 24.VII.2015, leg. Dan Xu; Yunnan: 2 ♂♂, Honghe Prefecture, Ma’andi, 1300 m, 14.V.2015, leg. Xue-Li Wei; 2 ♂♂, Xishuangbannadaizu Prefecture, Menglun Town, 620 m, 17.V.2018, leg. Xiao-Qiang Lu & Xi-Cui Du; Guangxi: 1 ♂, Longzhou, Nonggang, 188 m, 26.VII.2011, leg. Gui-Qing He; 1 ♂, Jingxiu, Shengtang Mountain, 600 m, 28.VIII. 2011, leg. Li-Yang Jun; 1 ♂, Jinzhong Mountain, Miaozhai, 1450 m, 31.VII.2014, leg. Xue-Li Wei & Chao Ran; 3 ♂♂, Cenwanglaoshan, Longdaping, 1290 m, 10.VIII.2014, leg. Xue-Li Wei & Chao Ran; 1♂, Hechi, Jiuwanshan, 1600 m, 23.VII.2015, leg. Ji-Ping Wan; Hainan: Wuzhi Mountain, 795 m, 20.V.2014, leg. Li-Jun Xu & Xu Dan. Genitalia slide no.: XLJ13114 ♂, XLJ14053 ♂, XLJ14219 ♂, LXQ18284 ♂, LXQ18291 ♂, LXQ18303 ♂, XLJ14220 ♀, XLJ14239 ♀.

##### Diagnosis.

Adult (Fig. 5): Wings brown. Forewing length 10.0–12.5 mm (wingspan 22.0–28.0 mm); a small white spot between the orbicular spot and discoidal spot; a large white subreniform spot between the discoidal spot and postmedial line, up to Rs_2_+Rs_3_ and down to CuA_1_; antemedial and postmedial line unclear. Hindwing with a large white irregular quadrilateral spot between the discoidal spot and postmedial line, dentated between M_2_ and M_3._ Male genitalia (Fig. 10): Uncus trapezoidal, distal half with setae. Gnathos stubby, fingerlike or tuberculiform. Clasper thin, fingerlike. Female genitalia (Fig. 13): Signum round, very small.

##### Male genitalia

(Fig. 10). Uncus trapezoidal, slightly concave terminally, distal half with setae. Gnathos stubby, finger-like or tubercle-like. Valva elongate lingulate, slightly narrowed, terminal with a crowd of long setae, posterior margin with a cluster of long setae near the middle and slightly concave distally; clasper thin, finger-like, constricted near middle. Saccus conical, broad. Juxta semi-circular. Phallus longitudinally wrinkled distally.

##### Female genitalia

(Fig. 13). Apophyses anteriores ca. twice the length of apophyses posteriores. Ductus bursae ca. twice the length of corpus bursae; ductus seminalis from the middle of ductus bursae. Corpus bursae oval, with a very small leaflike signum.

##### Distribution.

China (Liaoning, Gansu, Shanxi, Shaanxi, Henan, Hubei, Zhejiang, Jiangsu, Jiangxi, Tibet, Sichuan, Chongqing, Guizhou, Yunnan, Guangdong, Guangxi, Hainan, Fujian, Taiwan), Korea, Japan, India, Russia (far east) (Hampson 1899; Inoue 1982; Du 2009).

#### Nagiella quadrimaculalis

Taxon classificationAnimaliaLepidopteraCrambidae

(Kollar & Redtenbacher, 1844)

F2D86EA8-E653-55FA-98E7-3EE7A737CF04

Figures 6 , 11 , 14

Scopula quadrimaculalis Kollar & Redtenbacher, 1844: 492.
Nagia desmialis Walker, 1866: 1320.
Omiodes quadrimaculalis : Meyrick, 1890: 441.
Botys quadrimaculalis : Snellen, 1890: 589.
Sylepta [sic] quadrimaculalis: Hampson, 1896: 336.
Sylepta [sic] desmialis: Swinhoe, 1906: 293.
Nagiella quadrimaculalis : Munroe, 1976: 876.
Pleuroptya quadrimaculalis : Inoue, 1982: 343.

##### Material examined.

China, Gansu: 4 ♂♂, 3 ♀♀, Kangxian County, Baiyun Mountain, 1200 m, 3.VII.2018, leg. Xiao-Qiang Lu & Xi-Cui Du; Shanxi: 4 ♂♂, Lishanxiachuan Nature Reserve, 1560 m, 26.VII.2012, leg. Gui-Qing He; Shaanxi: 11 ♂♂, Ningshan County, Yangjuba Town, 1400 m, 4.VIII.2014, leg. Hai-Li Yu & Jiu-Yang Luo; 7 ♂♂, 8 ♀♀, Yang County, 3500 m, 15.VIII.2017, leg. Jian-Yue Qiu & Hao Xu; 7 ♂♂, 1 ♀, Taibai County, Huangbaiyuan, 1291 m, 16.VII.2018, leg. Qing-Ming Liu; Henan: 33 ♂♂, Neixiangbaotianman Nature Reserve, Luotiofeng, 1300 m, 8.VI.2017, leg. Jian-Yue Qiu & Hao Xu; Hubei: 28 ♂♂, 3 ♀, Dabie Mountain, Taohua Village, 590 m, 20.VII.2010, leg. Li-Jun Xu; 4 ♂♂, Luotian County, Qingguantai, 580 m, 1.VII.2014, leg. Jiu-Yang Luo; 11 ♂♂, Changyang County, Hejiaping, 800 m, 18.VI.2018, leg. Xiao-Qiang Lu & Xi-Cui Du; 4 ♂♂, Wufenghou River, 1100 m, 26, VII.2018, leg. Jian-Yue Qiu & Hao Xu; Hunan: 7 ♂♂, Shimen County, Huping Mountain, Dadongping, 1400 m, 8.VI.2017, leg. Jian-Yue Qiu & Hao Xu; 5 ♂♂, Sangzhi County, tianping Mountain, 1400 m, 15.VII.2018, leg. Jian-Yue Qiu & Hao Xu; 6 ♂♂, 2 ♀♀, Yizhang County, Mangshan Nature Reserve, 1000 m, 2.VIII.2018, leg. Jian-Yue Qiu & Hao Xu; Zhejiang: 10 ♂♂, Tianmu Mountain Nature Reserve, 400 m, 26–29.VII.2011, leg. Xiao-Bing Fu & Xi-Cui Du; 11 ♂♂, 3 ♀♀, Qingliang Mountain, Shunxiwu, 300 m, 18–21.V.2012, leg. Xiao-Bing Fu; Jiangxi: 1 ♂, 1 ♀, Jinggangshan City, Xiaoxidong, 625 m, 30.V.2011, leg. Jin-Wei Li; Chongqing: 18 ♂♂, 10 ♀♀, Jingfo Mountain Nature Reserve, 1700 m, 12.VII.2010, leg. Xi-Cui Du & Sheng-wen Shi; 10 ♂♂, Simian Mountain Nature Reserve, 1120 m, 19.VII. 2010, leg. Xi-Cui Du & Li-fang Song; 3 ♂♂, Simian Mountain Nature Reserve, 1200 m, 15–19. VII. 2012, leg. Gui-Qing He & Li-Jun Xu; 13 ♂♂, 1 ♀, Chengkou County, Xingtian Village, 1300 m, 1.VII.2013, leg. Gui-Qing He & Li-Jun Xu; Sichuan: 4 ♂♂, 1 ♀, Luding County, Hailuogou, 3478 m, 4.VII.2012, leg. Jin-Wei Li; 19 ♂♂, Nanjiang County, Guangwu Mountain, 700 m, 3.VII.2013, leg. Gui-Qing He & Li-Jun Xu; 21 ♂♂, 14 ♀♀, Xuyong County, huagaoxi Nature Reserve, 621 m, 26–30.VIII.2013, leg. Dan Xu & Xue-Li Wei; 19 ♂♂, 3 ♀♀, An’zi River Nature Reserve, 1690 m, 4.VIII.2015, leg. Xi-Cui Du; Guizhou: 11 ♂♂, 1 ♀, Kuankuoshui Nature Reserve, 800 m, 10–17.VIII.2010, leg. Xi-Cui Du; 5 ♂♂, Libo County, An’xiang, 1345 m, 22.VII.2015, leg. Ji-Ping Wan; Yunnan: 1 ♂, 1 ♀, Ninglang County, Xichuan, 2400 m, 20.VII.2013, leg. Gui-Qing He; 19 ♂♂, 2 ♂♂, Malipo County, Daxichang, 1465 m, 7.VI.2015, leg. Man-Fei Tao; Dawei Mountain Nature Reserve, 2700 m, 27.V.2018, leg. Xiao-Qiang Lu & Xi-Cui Du; 10 ♂♂, Huanglian Mountain Nature Reserve, 900 m, 23.VI.2018, leg. Xiao-Qiang Lu & Xi-Cui Du; 15 ♂♂, 1 ♀, Xihuangbanna Prefecture, Menglun Town, 620 m, 17.V.2018, leg. Xiao-Qiang Lu & Xi-Cui Du; Guangdong: 27 ♂♂, 15 ♀♀, Nanlingbabao Mountain Nature Reserve, 1070 m, 22.VIII.2010, leg. Xi-Cui Du; 2 ♂♂, 1 ♀, Shixing County, Baling Nature Reserve, 496 m, 29.V.2017, leg. Yong-Hong Duan (SYSU); Guangxi: 22 ♂♂, Hechi City, Jiuwan Mountain, 1600 m, 26.VII.2015, leg. Ji-Ping Wan; 4 ♂♂, Guilin City, Maoer Mountain Nature Reserve, 1100 m, 23.VII.2015, leg. Kai-Li Liu & Jing-Xia Zhao; 9 ♂♂, Rongshui County, Peixiu Village, 1900 m, 24.VIII.2015, leg. Ji-Ping Wan; 12 ♂♂, Cenwanglao Mountain, Dalongping, 1290 m, 4.VIII.2014, leg. Xue-Li Wei & Chao Ran; Fujian: 8 ♂♂, Wuyi Mountain Nature Reserve, Tongmu Village, 758 m, 20.VIII.2016, leg. Kai Chen & Yong-Hong Duan (SYSU). Genitalia slide no.: XLJ13123 ♂, XLJ13158 ♂, XLJ13215 ♂, XLJ14029 ♂, XLJ14056 ♂, XLJ14075 ♂, XLJ14076 ♂, XLJ14133 ♂, XLJ14229 ♂, LXQ19304 ♂, LXQ19305 ♂, LXQ18308 ♂, LXQ18310 ♂, XLJ13124 ♀, XLJ13159 ♀, XLJ13216 ♀, XLJ114012 ♀, XLJ14030 ♀, LXQ18306 ♀.

##### Diagnosis.

Adult (Fig. 6): Wings brown. Forewing length 12.0–20.0mm (wingspan 26.0–43.0 mm); a small white spot between the orbicular spot and discoidal spot; a large white sub-reniform spot between the discoidal spot and postmedial line, up to Rs_2_+Rs_3_ and down to CuA_1_; antemedial and postmedial line unclear. Hindwing with a large white irregular quadrilateral spot between the discoidal spot and postmedial line, dentated between M_2_ and M_3._ Male genitalia (Fig. 11): Uncus trapezoidal. Gnathos slender, finger-like. Clasper thickly finger-like. Female genitalia (Fig. 14): Signum small, round.

##### Male genitalia

(Fig. 11). Uncus trapezoidal. Gnathos slender, finger-like. Valva elongate lingulate, with apex narrowed, posterior margin with a cluster of long setae near the middle; clasper thickly finger-like. Saccus conical, broad. Juxta peach-shaped. Phallus longitudinally wrinkled distally.

##### Female genitalia

(Fig. 14). Apophyses anteriores ca. twice the length of apophyses posteriores. Ductus bursae ca. twice the length of corpus bursae, distinctly narrowed near the base; ductus seminalis from approximately one third of the ductus bursae. Corpus bursae oval, with a small round signum.

##### Distribution.

China (Heilongjiang, Liaoning, Gansu, Shanxi, Shaanxi, Henan, Hebei, Hubei, Shandong, Hunan, Zhejiang, Jiangxi, Tibet, Sichuan, Chongqing, Guizhou, Yunnan, Guangdong, Guangxi, Hainan, Fujian, Taiwan), Korea, Japan, Indonesia, India (Sikkim), Nepal, Russia (far east), Malaysia (Walker 1866; Inoue 1982; Du 2009).

##### Host.

*Rhus chinensis* Mill (Anacardiaceae) (Fan and Piao 2013).

##### Remarks.

In addition to *Rhus chinensis* Mill, another host, *Metaplexis japonica* Makino (Apocynaceae), was recorded by Fan and Piao (2013) in the same article according to Yoshiyasu (1991). However, we found *M. japonica* was recorded by Yoshiyasu (1991) as the host of *Glyphodes quadrimaculalis* (Bremer and Grey 1853) but not of *N. quadrimaculalis* (Kollar and Redtenbacher). *Rhus chinensis* Mill is the only host of *N. quadrimaculalis* (Kollar and Redtenbacher) known so far.

Swinhoe (1894) stated that *Nagia incomitata* was between *Nagia quadrimaculalis* and *N. flavispila*, but quite different to either. But *N. incomitata* was regarded as a synonym of *N. quadrimaculalis* because they were similar in habitus (Bae et al. 2008). We investigated the original description and type specimen of *N. incomitata* Swinhoe, 1894, and compared them with the description and photographs of *N. quadrimaculalis* (Kollar and Redtenbacher 1844; Du 2009; Sasaki and Yamanaka 2013). The third segment of labial palpus of the former is slender and pointed distally (Fig. 7A), the forewing has no small white spot between the orbicular spot and discoidal spot, and the large white spot beyond the cell is down to the CuA_2_ (Fig. 7); while the third segment of labial palpus of the latter is stubby and blunt distally (Fig. 2), the forewing has a small white spot between the orbicular spot and discoidal spot, and the large white spot beyond the cell is down to the CuA_1_ (Fig. 6). Therefore, *N. incomitata* is not a synonym of *N. quadrimaculalis*. *Nagia incomitata* was transferred to *Chalcidoptera* Butler, 1887 by Swinhoe (1901) after stating previously that it did not belong into *Nagia* (Swinhoe 1900). Hampson (1896), on the other hand, considered it a synonym of *Nosophora chironalis* (Walker, 1859), which he later revised (Hampson 1903) by reinstating it as *Nosophora incomitata*, with the junior synonym *Nosophora triguttalis* Warren, 1896. In the same publication on page 216, Hampson (1903) synonymised the males of *N. incomitata* with *Sylepta* [sic] *quadrimaculalis*. For the time being (i.e., until the type material has been investigated), we conclude as Hampson (1903), Klima (1939b), and Mandal and Bhattacharya (1980), who considered *incomitata* a species of *Nosophora*.

#### Nagiella occultalis

Taxon classificationAnimaliaLepidopteraCrambidae

Misbah & Yang in Ullah et al. 2017

9CC5656C-8F4F-5E8E-8B25-E7A67A4A4F3F

Nagiella occultalis Misbah & Yang in Ullah et al. 2017: 70. Figs 2A, 3, 4A, B.

##### Note.

Description of the habitus and genitalia was provided by Ullah et al. (2017).

##### Distribution.

China (Shaanxi, Hubei) (Ullah et al. 2017).

#### Nagiella bispina sp. nov.

Taxon classificationAnimaliaLepidopteraCrambidae

7E56D304-3F9B-50DA-B4E1-03685AB576D3

http://zoobank.org/EA3EDE34-1DEA-4FA7-B5A9-70F596B3B2DE

Figures 8 , 12 , 15 , 15A

##### Type material.

***Holotype*.** ♂, pinned, with genitalia on a separate slide. China, Guangdong: Nanling, Babao Mountain Nature Reserve, 24.98N, 113.03E, 1070 m, 23.VIII.2010, leg. Xi-Cui Du, genitalia slide no. XLJ14011 ♂. ***Paratypes*.** China, Guangdong: 1 ♂, 1 ♀, same data as holotype. Genitalia slide no.: XLJ14009 ♀, XLJ14134 ♂.

##### Diagnosis.

This species is very similar to *N. quadrimaculalis* externally, but can be distinguished from the latter by its rather short and wide uncus with distal margin round, gnathos absent, clasper thick thorn-like, phallus with a hook-shaped cornutus; ductus bursae ca. the same length as corpus bursae with two thorn-like signa (Fig. 15A). In *N. quadrimaculalis*, the uncus is trapezoidal, gnathos is slender and finger-like, clasper is thickly finger-like, and phallus exhibits no cornuti; ductus bursae is ca. twice the length of corpus bursae and corpus bursae has a small round signum (Fig. 14).

##### Description.

Adult (Fig. 8). Body brown tinged with copper-colour. Forewing length 11.5–13.5 mm (wingspan 26.0–30.0 mm). Frons, vertex, antenna and maxillary palpus brown. Male antenna with ventral cilia ca. half as long as the diameter of flagellomere. Labial palpus with first and second segments white ventrally, the rest brown. Thorax and abdomen brown dorsally, off-white ventrally. Legs off-white, fore tibia brown distally. Wings brown. Forewing with antemedial line excurved, unclear; orbicular spot and discoidal spot dark brown, the latter squarish; a small white spot between the orbicular spot and discoidal spot; a large white sub-reniform spot between the discoidal spot and postmedial line, up to Rs_2_+Rs_3_ and down to CuA_1_; postmedial line unclear, from ca. 2/3 of the costa, along outer edge of the large white spot, excurved from M_2_ to CuA_2_, then incurved and nearly vertical to the inner margin below the posterior angle of cell; cilia lightly brown with white basal line. Hindwing with discoidal spot dark brown, short band; a large white irregular quadrilateral spot between the discoidal spot and postmedial line, dentated between M_2_ and M_3_; postmedial line unclear, along outer edge of the large white spot, lightly excurved from M_2_ to CuA_2_, then incurved and nearly vertical to the inner margin below the posterior angle of cell; cilia lightly brown with white basal line. Abdomen with each segment white distally.

##### Male genitalia

(Fig. 12). Uncus rather short and wide, with distal margin round. Gnathos absent. Valva lingulate, slightly widened; clasper thick thorn-like, with a cluster of long setae at the base. Saccus conical. Juxta near diamond. Phallus with a thick hook-shaped cornutus.

##### Female genitalia

(Fig. 15, 15A). Apophyses anteriores ca. twice the length of apophyses posteriores. Ductus bursae ca. the same length as corpus bursae, expanded and sclerotized near the middle; antrum slightly sclerotized; ductus seminalis from expanded part. Corpus bursae oval; two thorn-like signa of different sizes, surrounded by dense microspines.

##### Etymology.

The specific name, *bispina*, is derived from the Latin *bi* (meaning two or double) and *spina* (meaning spine or thorn) in reference to the two thorn-like signa.

##### Distribution.

China (Guangdong).

## Supplementary Material

XML Treatment for Nagiella

XML Treatment for Nagiella hortulatoides

XML Treatment for Nagiella inferior

XML Treatment for Nagiella quadrimaculalis

XML Treatment for Nagiella occultalis

XML Treatment for Nagiella bispina
